# Hepatocellular Carcinoma: The Search for an Optimal Screening Test

**DOI:** 10.34172/mejdd.2025.407

**Published:** 2025-01-31

**Authors:** Sara Haj Ali, Shahd I Alqato, Amjad M Almansi, Noor S Haj Ali, Mohammad A Amaireh

**Affiliations:** ^1^Internal Medicine Department, Faculty of Medicine, Al-Balqa Applied University, Salt 19117, Jordan; ^2^Internal Medicine Department, Arab Medical Center, Amman 11181, Jordan; ^3^Al-Saudi Hospital, Amman 11953, Jordan

**Keywords:** Hepatocellular carcinoma, Screening, Alpha-fetoprotein, Liquid biopsy, Surveillance

## Abstract

Hepatocellular carcinoma (HCC) is the sixth most common cancer worldwide and the third leading cause of cancer-related death, with a 5-year survival rate of 10%-12%. It usually develops in the setting of chronic liver disease (CLD), with chronic viral hepatitis, alcohol, and non-alcoholic fatty liver disease (NAFLD) being the most common risk factors. Some patients are at higher risk of developing hepatocellular cancer, so it is important to screen them regularly to diagnose the disease at an early stage and improve their chances for curative treatment. Six-monthly ultrasound with or without alpha-fetoprotein (AFP) is the currently recommended surveillance method. AFP has been used as a biomarker for liver cancer; however, it has low sensitivity and specificity, which necessitates the search for other, more accurate biomarkers. Promising biomarkers include lens culinaris agglutinin-reactive AFP, des-gamma-carboxy prothrombin, methylated DNA markers, plasma microRNA expression, circulating tumor DNA, and circulating tumor cells. In addition, combinations of biomarkers, like the GALAD score and the Doylestown algorithm, may help in the early detection of HCC. In this review, we summarize the screening tests for early detection of HCC that have been studied over the last decade.

## Introduction

 Hepatocellular carcinoma (HCC) constitutes over 80% of primary liver cancers. It ranks as the sixth most common cancer worldwide and the third leading cause of cancer-related mortality, as reported by the World Health Organization Global Cancer Observatory (GLOBOCAN) 2020.^1^ The HCC incidence varies across different regions, with over 80% of cases occurring in East Asia and sub-Saharan Africa.^2^

 The incidence of HCC has increased in most European countries, North America, and Australia while decreasing in many Asian countries.^3^ In the United States, the incidence of HCC has increased by two- to three-fold due to the high prevalence of hepatitis C virus (HCV), non-alcoholic fatty liver disease (NAFLD), and metabolic syndrome.^2^ The incidence of HCC among patients with chronic viral hepatitis has decreased since the introduction of direct-acting antiviral treatment for HCV and hepatitis B virus (HBV) vaccination.^4^

 HCC is more common in men than women in most countries, including the United States, and among certain ethnic groups such as Hispanics, African Americans, Whites, and Asians.^5^ Moreover, HCC often occurs in older patients in many countries compared to Africa.^2^ Worldwide, 80% of HCC cases occur in patients with HBV and HCV infections.^2^ Additionally, the risk of HCC is further increased in patients co-infected with other viruses like human immunodeficiency virus (HIV) and hepatitis D virus (HDV).^5^ Other risk factors for HCC include smoking, diabetes, a family history of HCC, obesity, heavy alcohol consumption, and aflatoxin exposure.^2^

 Several genetic and metabolic disorders also increase the risk of developing HCC. For example, patients with NAFLD are at a higher risk of developing HCC compared with the general population. Similarly, other disorders like hereditary hemochromatosis, alpha-1 antitrypsin deficiency, and glycogen storage diseases also represent a smaller proportion of HCC cases globally compared with viral hepatitis.^2^

 The long-term outcome of HCC is poor, with a 5-year survival rate of only 10%–12%.^6^ Implementing strict surveillance programs improves early detection of HCC and increases overall survival. Studies have indicated a decrease in the mortality rate of HCC in patients who underwent six-monthly surveillance compared with those who did not (83 versus 132 per 100 000).^7^ Additionally, a meta-analysis has shown that surveillance increases the chance of diagnosing HCC in early stages, thus increasing the opportunity of curative therapy and improving the outcome.^8^

 Surveillance must target patients at a high risk of developing HCC (Box 1). NAFLD is increasingly becoming a prevalent cause of chronic liver disease (CLD). However, despite its rising burden, patients with NAFLD are less likely to receive surveillance compared with those with other causes of HCC.^9^ The American Association for the Study of Liver Diseases (AASLD) recommends a 6-monthly ultrasound with alpha-fetoprotein (AFP) to screen for HCC in high-risk patients eligible for curative treatments. However, patients with compromised liver function, including those with Child-Pugh C cirrhosis, are excluded from surveillance due to low survival rates unless placed on the transplant list.^10^

**Box 1. High-risk patients who need HCC surveillance**

Patients with Child-Pugh class A or B cirrhosis of any etiology

Patients with Child-Pugh class C awaiting liver transplantation

Patients with non-cirrhotic chronic hepatitis B:

- Males from endemic countries with age ≥ 40 years
- Females from endemic countries with age ≥ 50 years
- Africans > 20 years
- Family history of HCC
- PAGE-B score ≥ 10^a^

Abbreviations: HCC, hepatocellular carcinoma

^a^PAGE-B is a risk prediction score for patients with chronic hepatitis B infection that consists of platelets, age, and gender. A score of 10-17 corresponds to intermediate risk for HCC, whereas a score of ≥ 18 corresponds to high risk for HCC.

Diagnosis is usually done non-invasively using contrast-enhanced imaging, like computed tomography scans or magnetic resonance imaging. Contrast-enhanced ultrasound has also been used to characterize liver lesions with comparable results.^11^ Based on imaging characteristics in high-risk patients, societal guidelines have developed diagnostic criteria for HCC with some variations between them. Liver biopsy is usually reserved for cases with indeterminate imaging results.

 There are two types of treatment in HCC: curative and non-curative. Curative treatments include resection, liver transplantation, and ablative therapies. Non-curative treatments include transarterial chemoembolization (TACE), transarterial radioembolization (TARE), and systemic chemotherapy. Treatment choice depends on the number and size of lesions, anatomic site, liver function, and portal pressure.^10^

 This review aimed to present the advancements in screening and early detection of HCC.

## Methods

 The PubMed search was done using keywords ‘hepatocellular carcinoma’, ‘HCC’, ‘screening’, ‘surveillance’, and ‘early detection’ for articles published in English between January 2013 and October 2023. Searched articles included original articles, meta-analyses, reviews, observational studies, and case series. Information regarding HCC screening and recent developments in early detection was collected.

## HCC Screening

 Currently, the most widely used method for HCC surveillance, as per societal guidelines, involves ultrasound, either alone or combined with AFP every 6 months.^12^ However, the accuracy and reliability of ultrasound examinations can be influenced by the ultrasound practitioner’s expertise and the equipment’s quality, potentially yielding less precise results compared with serological indicators. A meta-analysis evaluating the performance of surveillance ultrasound with and without AFP revealed that ultrasound sensitivity for early HCC detection increased from 45% (95% CI: 30%–62%) when used alone to 63% (95% CI: 48%–75%) when combined with AFP.^13^ Consequently, the effectiveness of these methods in screening and diagnosing small and early-stage HCC remains insufficient regarding both sensitivity and specificity.^14^ There is an urgent need to investigate novel approaches to identify high-risk populations for liver cancer and screen for patients with HCC, particularly those with early-stage HCC, AFP-negative HCC, and subclinical micro-liver cancer.^15^

 Numerous newly studied biomarkers have shown promising results, and they include a more specific subfraction of AFP—lectin-reactive AFP (AFP-L3)—and des-gamma-carboxy prothrombin (DCP), alongside novel cancer biomarkers and assays for hypermethylation and DNA mutations. Moreover, diagnostic models that use some of the recently studied biomarkers have also been evaluated.

## Biomarkers

###  Alpha-Fetoprotein 

AFP is the most commonly used biomarker for HCC. However, its use as a surveillance test is controversial per societal guidelines due to inadequate sensitivity and specificity. It has 46%–59% sensitivity for clinical HCC diagnosis and only 40% for preclinical prediction.^16,17^ Moreover, approximately 30% of patients with liver cancer consistently exhibit negative serum AFP levels.^18^ Additionally, AFP levels may correlate with alanine transaminase (ALT) levels, thereby limiting its specificity, especially in cases of active liver inflammation.^19^ A cutoff value of 20 ng/mL yields a 60% sensitivity and 90% specificity for early detection of HCC.^20^ Some studies have indicated that changes in AFP value over time may increase its sensitivity and specificity compared with a single AFP value.^21^

 A study was conducted to evaluate how well AFP, AFP-L3, DCP, and various combinations of these markers could detect HCC in at-risk patients. Using samples collected over time from respective patients, the study found that among the three biomarkers, AFP demonstrated the most effective performance in distinguishing between HCC cases and control subjects with an area under the curve (AUC) of 0.77 at the time of diagnosis compared with an AUC of 0.73 for AFP-L3 and 0.71 for DCP. Similarly, AFP had a sensitivity of 62% compared with 55% for AFP-L3 and 48% for DCP.^22^

###  Lens Culinaris Agglutinin-Reactive Fraction of Fetoprotein (AFP-L3)

 A subtype of AFP, known as the lens culinaris agglutinin-reactive fraction of fetoprotein (AFP-L3), originates from malignant hepatocytes and is considered a specific marker for HCC.^23^ A meta-analysis that evaluated the diagnostic potential of AFP-L3 in detecting HCC revealed that AFP-L3 exhibited strong diagnostic capability for HCC with 70% sensitivity and 91% specificity, and the sensitivity was even higher (79%) in the Asian population.^24^

###  Des-Gamma-Carboxy Prothrombin /Prothrombin Induced by Vitamin K Absence or Antagonist-II 

 Prothrombin induced by vitamin K absence or antagonist-II (PIVKA-II), also known as DCP, is an abnormal prothrombin protein that is elevated in the blood of patients with HCC.^25^ The production of PIVKA-II is believed to stem from a malfunction in the post-translational carboxylation of the prothrombin precursor within cancerous cells. Serum PIVKA-II levels were notably elevated in patients with HCC compared with those with benign liver conditions and healthy controls.^26^

 A study evaluating the diagnostic performance of PIVKA-II as a standalone biomarker for identifying HCC revealed outstanding results. PIVKA-II demonstrated substantial diagnostic utility even in cases of AFP-negative HCC. Significant associations were observed between PIVKA-II expression levels and various clinicopathological characteristics, including tumor size, stage, metastasis, differentiation status, and associated complications. After surgical removal of the tumor, PIVKA-II expression markedly decreased, indicating its value in assessing the success of HCC resection.^26^

 A retrospective study that investigated the effectiveness of serum DCP in the diagnosis of AFP-negative HCC in patients with HBV infection revealed that DCP had a relatively strong ability to distinguish between AFP-negative HBV-related HCC and chronic HBV infection or liver cirrhosis.^27^ This result was consistent with a larger multicenter study, which showed that DCP was superior to AFP for the surveillance of early AFP-negative HCC.^28^ Therefore, DCP could be a promising biomarker to improve the early detection of AFP-negative HBV-related HCC.

 DCP may have a lower discriminatory ability than AFP in distinguishing early-stage HCC in patients with chronic HBV infection from those with chronic hepatitis B.^29^

## Combinations of Biomarkers

###  AFP and DCP/PIVKA-II

 PIVKA-II is a promising serum biomarker for the diagnosis of HCC and can serve as an important complement to AFP. Utilizing both markers for diagnosis significantly improves the overall diagnostic effectiveness of HCC.^30^ The AFP and PIVKA-II are crucial in diagnosing HBV-related HCC. The diagnostic utility of combining the detection of AFP and PIVKA-II or using PIVKA-II as a single assay surpasses the effectiveness of separately assessing AFP. Additionally, the concentration of these biomarkers holds significant clinical value in determining aspects, including tumor size, tumor cell differentiation, and vascular invasion.^31^

 For differentiating liver cirrhosis-related HCC from liver cirrhosis, both AFP and DCP exhibited similar diagnostic performance. However, the combined use of AFP and DCP did not significantly enhance diagnostic accuracy.^29^ In a pilot clinical trial cohort, a diagnostic model incorporating AFP, PIVKA-II, age, and sex accurately predicted HCC occurrence in high-risk patients with HBV. While some variables in this nomogram overlapped with the GALAD score for patients with chronic HBV, it was observed that AFP-L3 did not perform as good as AFP or PIVKA-II in terms of diagnostic accuracy. Furthermore, AFP-L3 did not show significant relevance in the multivariable model. These findings suggest that the AFP-L3 marker may be unsuitable for HCC detection in patients with chronic HBV, leading to its exclusion from the final nomogram.^32^

 PIVKA-II was superior to AFP for HCC screening in Chinese patients, and it may predict the prognosis of patients through its correlation with the risk of portal vein tumor thrombosis. When AFP and PIVKA-II were employed together, they substantially enhanced the diagnostic accuracy for the entire HCC cohort and those in the early-stage group.^33^

###  AFP and AFP-L3

 A study evaluating the effectiveness of AFP, AFP-L3, and DCP and their combinations for early HCC detection utilized longitudinally collected samples from at-risk patients. The analysis revealed that combining AFP and AFP-L3 using cutoff values of 5 ng/mL and 4%, respectively, significantly enhanced the sensitivity (from 62% to 79%) for detecting very early-stage HCC.^22^

###  Triple Combination (AFP, AFP-L3, and DCP)

 The combined sensitivity and specificity of AFP, AFP-L3, and DCP were 88% and 79%, respectively, surpassing the sensitivity (63%; 95% CI: 48%–75%) of the current surveillance method for early-stage HCC detection, which involves ultrasound with AFP.^34^ These findings imply that combining AFP, AFP-L3, and DCP offers high overall diagnostic performance and can benefit HCC diagnosis and screening. This aligns with the results of another meta-analysis, which indicated that the combined triple-panel of AFP, AFP-L3, and DCP demonstrated higher diagnostic performance compared with individual or random double combinations of these three biomarkers.^30^

 A study evaluating the combined and individual abilities of AFP, AFP-L3, and DCP in distinguishing between early HBV-related HCC and liver cirrhosis revealed that AFP and DCP had the highest AUC values for distinguishing between these two conditions.^35^ Interestingly, including AFP-L3 did not enhance the diagnostic performance compared with the combination of AFP and DCP. These findings suggest that combining AFP and DCP may be the most cost-effective approach for monitoring patients for HCC.

###  Liquid Biopsy

 As many tumors do not demonstrate high AFP levels and imaging techniques have low sensitivity, especially for smaller tumors or cirrhosis, alongside advancements in our understanding of the molecular mechanisms behind HCC, there is an increasing demand for molecular data about these tumors. While biopsies provide valuable information, they are invasive and may be impractical due to tumor location. In this setting, the emergence of liquid biopsy technology presents a promising avenue for early diagnosis, molecular characterization, disease monitoring, and predicting prognosis.^36^ The concept of “liquid biopsy” was first introduced as an innovative diagnostic approach in 2010 to examine circulating tumor cells (CTCs) in the blood of patients with cancer.^37^ Since then, it has been expanded to include other applications like examining circulating tumor DNA, extracellular vesicles, tumor-educated platelets, and circulating RNA in various body fluids.^38^ The liquid biopsy is precise, and leveraging next-generation sequencing and multi-omics technologies increases the sensitivity of the results, which are essential in HCC, which is known to have a high degree of heterogeneity and various molecular subtypes.^39^

###  Circulating Tumor Cells 

 Circulating tumor cells (CTCs), first described in 1869, constitute a small fraction of cells that leave solid tumor lesions and enter the bloodstream, surviving for a short time.^39,40^ Even though they play a key role in the initiation of metastasis, they can also be found in the early stages of cancer. They are unique from other liquid biopsy markers by serving as a definitive indication of the presence of viable tumors, even in situations where conventional imaging modalities fall short in their detection.^41^ CTCs are being investigated for their potential applications in early diagnosis, prognosis, and the monitoring of treatment responses in various types of tumors, including HCC.^40^ It has been shown that their number correlates with the HCC stage and AFP level.^42^ Moreover, CTCs provide valuable insights into the characteristics of tumors, offering information about multiple molecular alterations, genetic mutations (the most common are *TP53*, *CTNNB1*, *TTN*, *MUC16*, and *ALB*), and DNA methylation changes (for example, hypermethylated genes *CDKN2A*, *RASSF1*, *APC*, and *SMAD6*).^43^ A meta-analysis that studied the diagnostic accuracy of CTCs for early diagnosis of HCC revealed very good results with AUC = 0.91, sensitivity of 0.95 (95%CI = 0.93–0.96) but with a specificity of 0.60 (95% CI = 0.57–0.63).^44^ Several studies have suggested combining CTCs with other biological markers like AFP can enhance their sensitivity and specificity in HCC diagnosis.^45,46^ Nonetheless, isolating CTCs from background cells poses challenges, and the phenotypic and genotypic heterogeneity complicates standardized detection methods.^44^

###  Circulating Tumor DNA and Cell-Free DNA 

 Both cell-free DNA (cfDNA) and circulating tumor DNA (ctDNA) are DNA fragments released by apoptotic and necrotic cells. The ctDNA is a highly variable fraction of tumor cell-derived cfDNA,^47^ harboring cancer-specific alterations in genetic and epigenetic aspects, including single nucleotide mutations, copy number aberrations, DNA methylation, and others.^48^ Although it is hard to conclude small studies measuring cfDNA concentrations in different settings, it has been shown that cfDNA concentration is variable in patients with cancer, from 0 up to more than 1000 ng/mL, compared with 0-100 ng/mL in healthy individuals.^49^ However, fluctuations in ctDNA levels are influenced by factors, including tumor type, location, and challenges associated with sample collection, rendering the establishment of a universal cutoff value for test positivity challenging.^50^ Furthermore, it has been observed that higher concentrations significantly correlated with tumor size, stage, and prognosis. Notably, compared with other cancer types, HCC had the highest concentrations.^51^ Despite its high specificity in identifying HCC (up to %88 for qualitative analysis), ctDNA demonstrated low sensitivity (down to 56% for qualitative analysis) and is currently not considered an independent tool for HCC detection. However, when combined with other biomarkers like AFP, ctDNA analysis exhibited higher diagnostic accuracy for early HCC detection and diagnosis.^48^ Therefore, integrating quantitative and qualitative ctDNA analysis with AFP assays can serve as a valuable approach.

###  DNA Methylation 

 Aberrant DNA methylation, including both hypo- and hypermethylation, is one of the epigenetic mechanisms involved in human cancer development, including HCC, as it can cause inactivation of multiple tumor suppressor genes.^52^ The identification and characterization of specific DNA methylation patterns in genes unique to tumor cells are commonly referred to as DNA methylation markers (DMMs). Notably, the methylation patterns exhibit considerable heterogeneity in HCC, largely attributed to the diverse risk factors associated with the disease, including HBV, HCV, alcohol consumption, and NAFLD.^53^ Examples of DMMs tested using cfDNA are HOXA1, EMX1, AK055957, ECE1, PFKP, and CLEC11.^54^ Furthermore, a recent study conducted on cfDNA utilized an algorithm incorporating 28 DMMs along with the tumor markers AFP, AFP‐L3, and DCP, as well as patients’ age and sex. The study revealed promising results and outperformed AFP alone and GALAD scores in early HCC detection; the test used was the HelioLiver Test.^55^ Using PCR techniques, researchers found elevated serum methylation levels of various tumor suppressor genes, including RASSF1A, COX2, and APC, in patients with HCC.^56,57^ Among these, serum hypermethylated RASSF1A was one of the most frequently detected epigenetic changes in HCC. It demonstrated a predictive value of 72.5% for early HCC diagnosis, particularly in patients with HCV infection,^58^ and exhibited higher sensitivity than AFP at a 20 ng/L cutoff value in patients with chronic HBV.^59^ Various other efforts have been invested in using these DMMs as novel biomarkers for early HCC detection. However, challenges like the limited sensitivity of these biomarkers in early tumor stages need to be addressed first. Nevertheless, the detection of DMMs in liquid biopsies will probably significantly impact HCC screening in the future.^53^

###  MicroRNA

 MicroRNAs (miRNAs) are a diverse class of noncoding RNA molecules that control post-transcriptional gene expression.^60^ The use of individual miRNAs for early HCC detection has been studied before, with a meta-analysis demonstrating high diagnostic accuracy for serum miRNAs that was not statistically different from AFP.^61^ However, combining miRNAs with AFP had better diagnostic accuracy than either alone (area under the curve of summary receiver operating characteristic (AUC-SROC) curve 0.94 [95% CI: 0.91-0.96]). Moreover, combinations of miRNAs for early detection of HCC have been studied. For example, the microRNA classifier comprising microRNA-29a, microRNA-29c, microRNA-133a, microRNA-143, microRNA-145, microRNA-192, and microRNA-505 demonstrated a higher sensitivity and better diagnostic ability for HCC than AFP in a wide spectrum of healthy individuals to patients with cirrhosis.^62^ In addition, combining multiple miRNAs to develop miRNA-based scores has shown promising accuracy in detecting HCC.^63^ These scores and biomarkers warrant further research to develop their clinical usage in the early detection and diagnosis of HCC.

## Viral Exposure Signature

 Various viruses entering the host can interact with each other or the host’s cells to shape the host’s immunity, potentially leaving molecular footprints. This interaction can alter the host immunological response, which may serve as a door to increase the susceptibility to early onset cancer development, whether those viruses stay or are cleared by the host immune defense.^64^ The VirScan technology was used to detect the exposure to 206 human viral species comprising over 1000 viral strains in patients with CLD, HCC, and healthy controls to test the association between viral exposure and disease development. A viral exposure signature (VES) of 61 viral strains was identified, including 18 strains that were positively associated with HCC, 11 of which were HCV. The VES detected was able to confidently differentiate patients with HCC from at-risk participants or healthy controls and demonstrated superior association with HCC than AFP and other clinical variables. Later, this was validated by a prospective cohort in the same study where it was capable of identifying patients with HCC 8.8 years before clinical diagnosis [AUC of 0.91 at baseline (95% CI: 0.87–0.96) and 0.98 at HCC diagnosis (95% CI: 0.97–1)].^65^ Those results imply that VES may be useful as a screening tool; however, further testing is required to evaluate its ability to reduce HCC mortality. Furthermore, its clinical value should be further compared to the existing HCC risk-prediction scores and tests.^66,67^

## GALAD Scoring System

 The GALAD scoring system is a serum-based, radiation-free, and non-invasive tool for detecting HCC. It was developed at a United Kingdom center in 2014 to test the probability of HCC in a patient with CLD. It involves five easily obtainable and cost-effective indices: gender (G), age (A), AFP-L3 (L), AFP (A), and DCP (D).^68,69^ The scoring system significantly outperformed and surpassed the low sensitivity of ultrasound and AFP alone in early HCC detection, up to 48 weeks before clinical diagnosis, regardless of the etiology, with an approximate overall sensitivity and specificity of 0.86 and 0.9, respectively.^68,70^ Among different etiologies, it had the highest sensitivity, with 10% higher sensitivity among HCV and non-viral liver disease populations than patients with HBV. Studies on the GALAD system in NAFLD-related HCC are scarce; one study on patients with non-alcoholic steatohepatitis revealed a high diagnostic accuracy with AUC of 0.94 in the non-cirrhotic population compared with 0.85 in the cirrhotic population.^71^ The performance of the score in patients with alcoholic liver disease or multiple etiologies is not yet established.^72^ The system has also been shown to be a good prognostic tool; it could classify patients into two groups of low and high risk of overall survival and recurrence, and this was best demonstrated in patients with well-ablated focal lesions.^69,70^ In a 12-year prospective study on patients with advanced liver disease, the diagnostic accuracy of GALAD remained unaffected by the addition of tests used in other scoring systems (albumin, bilirubin, and platelets).^73^ However, combining ultrasound and GALAD increased the diagnostic accuracy of GALAD for the detection of early HCC with an AUC of 0.97.^74^ According to the British data, the proposed cutoff value for the system is -0.63; however, this value is diverse across populations, which causes some limitations in generalizing the algorithm to a certain point.^72^

## Doylestown Algorithm

 In 2016, the Doylestown algorithm was developed using log-transformed AFP values with four other clinical values, including age, gender, alkaline phosphatase (ALP), and ALT levels. The following formula:


$$p=\frac{1}{1+\exp\left(n\left[10.307+(0.097*age)+(1.645*gender)+(2.315*\log\;AFP)+(0.011*ALP)+(n0.008*ALT)\right]\right)}$$

A cutoff value of 0.5 for HCC diagnosis was proposed and externally validated. At a fixed specificity of 95%, the algorithm improved the detection rate of HCC from 2% to 20% compared with AFP, and the area under the receiver operating characteristic curve (AUROC) of AFP increased from 4% to 12%; the benefit was equal regardless of the tumor size or the etiology of liver disease.^75^ Another study showed that although the specificity of the algorithm was similar to or slightly lower than AFP at a cutoff value of 20 ng/mL, it increased the biomarker performance in early HCC detection by 21% 0.5–1 year before diagnosis and up to 13% within 1–1.5 years of diagnosis. Consequently, the algorithm was deemed fit for preliminary screening and reducing misdiagnosed cases of HCC.^76^ Notably, Chinese patients seem to have lower algorithm values than Caucasians, indicating that the cutoff point for the score may require adjustment across populations. An algorithm modified by adding fucosylated kininogen was developed to help detect HCC in the early stages and in cases where AFP is negative.^77^ It has shown promising results; however, it still needs external validation.

 A summary of the screening tests for HCC comparing their performance is shown in Table 1.

**Table 1:** Performance of various tests for early HCC detection

**Test**	**Sensitivity (95% CI)**	**Specificity (95% CI)**	**Notes **
Ultrasound alone^78^	45%	94%	
Ultrasound + AFP^7^	63%	92%	
AFP^20^	60%	90%	Cutoff 20 ng/mL
AFP-L3^24^	70%	91%	
PIVKA-II^26^	84%	92%	
AFP + AFP-L3^22^	79%	87%	Cutoff 5ng/mL & 4%
AFP + AFP-L3 + DCP^34^	88%	79%	
CTCs^44^	95%	60%	
CtDNA^48^	56%	88%	Qualitative analysis
RASSF1A^58^	75%	80%	
DNA methylation markers using HelioLiver test^55^	76%	91%	
miRNA^61^	82%	85%	
miRNA + AFP^61^	87%	88%	
GALAD score^70^	85%	73%	

## Models for HCC Risk Stratification

 The aim of these models is to individualize the patient’s risk for developing HCC based on certain characteristics. They have been validated in some populations but still need further validation before application in clinical practice. The most widely studied model is PAGE-B, which consists of platelets count, age, and gender in patients with chronic hepatitis B infection. It stratifies patients into low-risk (score ≤ 9), intermediate-risk (score 10-17), and high-risk groups (score ≥ 18). PAGE-B and its modification mPAGE-B (with the addition of albumin) have been validated in treated Caucasian and Asian patientswith high negative predictive value for low-risk patients ranging between 98.1%-100%.^79,80^

## The role of Contrast-Enhanced Imaging in HCC Screening

 The use of a contrast-enhanced CT scan or MRI for screening of HCC is not recommended despite superior sensitivity due to concerns regarding radiation and contrast exposure in the case of a CT scan. Additionally, MRI’s cost-effectiveness, availability, and patient tolerability should be considered. These imaging modalities may serve as better alternatives compared with ultrasound in patients with obesity, those with steatotic liver disease, or significant liver heterogeneity. An abbreviated MRI scan that is shorter and more cost-effective than conventional MRI has shown better performance than surveillance ultrasound in small-sized studies.^81^ Contrast-enhanced ultrasound is commonly used in Europe to diagnose HCC as it helps characterize lesions that have been identified on conventional non-contrast ultrasound. However, it cannot be used as a standalone method for screening. Its diagnostic performance is comparable to CT scans and MRI, with a sensitivity of 78% and a specificity of 94%.^11^

## Conclusion

 HCC is among the major causes of cancer-related mortality, and its incidence rate has been rising worldwide. Surveillance has been shown to decrease the mortality of HCC as it allows early cancer detection. Several novel biomarkers have been introduced with promising results at initial stages that require further testing and external validation. So far, combining more than one biomarker seems to yield the highest accuracy.
